# KEGG-Based Functional Signatures Complement Taxonomic Profiles Associated with Spontaneous Decolonisation of Carbapenem-Resistant *Enterobacterales*

**DOI:** 10.3390/ijms27167092

**Published:** 2026-08-07

**Authors:** Olalla Lima, Nahir Rodríguez-Costas, Maria Teresa Pérez-Rodríguez, Carlos Davina-Nunez, Marta Represa, Pablo Rubiñán, Maximiliano Alvarez, Marina Ávila-Nuñez, Anton Filgueira, Clara Portela, Bernardo Sopeña, Francisco J. Vasallo Vidal, Sonia Pérez-Castro

**Affiliations:** 1 Infectious Diseases Unit, Internal Medicine Department, Complexo Hospitalario Universitario de Pontevedra, 36071 Pontevedra, Spain; martarepresa@gmail.com; 2 Infectious Diseases Research Group, Galicia Sur Health Research Institute (IIS Galicia Sur), SERGAS-UVIGO, 36213 Vigo, Spain; perezrodriguezmt@gmail.com (M.T.P.-R.);; 3 Medicine Department, University of Santiago de Compostela, 15705 Santiago, Spain; 4 Microbiology and Infectology Group, Galicia Sur Health Research Institute (IIS Galicia Sur), SERGAS-UVIGO, 36312 Vigo, Spain; nahir.rodriguez@iisgaliciasur.es (N.R.-C.); francisco.jose.vasallo.vidal@sergas.es (F.J.V.V.); 5 Microbiology Department, University of Vigo, 36310 Vigo, Spain; 6 Infectious Diseases Unit, Internal Medicine Department, Complexo Hospitalario Universitario de Vigo, 36312 Vigo, Spainclaraportelapino@gmail.com (C.P.); 7 Microbiology Department, Complexo Hospitalario Universitario de Vigo (CHUVI), SERGAS-UVIGO, 36312 Vigo, Spain; 8 Vascular Surgery Department, Complexo Hospitalario Universitario de Pontevedra, 36071 Pontevedra, Spain; 9 Internal Medicine Department, Complexo Hospitalario Universitario de Santiago, 15706 Santiago, Spain

**Keywords:** metabolic pathways, gut carriage, microbiota, OXA-48-type, carbapenem-resistant Enterobacterales

## Abstract

Understanding the functional potential of the gut microbiota for carbapenem-resistant Enterobacterales (CRE) decolonisation is essential for developing novel non-antibiotic strategies to promote their clearance. In a previous study, we identified distinct taxonomic signatures associated with spontaneous CRE decolonisation (DeCol). Here, we aimed to determine whether these taxonomic differences were accompanied by differences in the predicted functional potential of the gut microbiota. Patients were identified from a database of individuals colonised with CRE. We performed Illumina shotgun metagenomic sequencing on 14 persistent CRE carriage (Col) and 23 DeCol patients with OXA-48-producing isolates. Bioinformatic analysis was performed using SqueezeMeta and differential abundance of functional and metabolic genes was assessed using DESeq2. Several antimicrobial resistance genes, including *bla*OXA-48, were underrepresented in DeCol patients. In contrast, DeCol patients showed an overrepresentation of genes associated with motility, regulated adhesion, short-chain fatty acid (SCFA)-related pathways and alternative carbohydrate metabolism. These orthologue enrichment patterns are consistent with functions previously linked to intestinal homeostasis in the literature. Conversely, Col patients exhibited an overrepresentation of genes associated with redox defence, biofilm formation and amino acid metabolism, suggesting distinct predicted functional profiles between persistent carriage and spontaneous decolonisation. Spontaneous CRE decolonisation was associated with distinct KEGG-based functional signatures and a lower abundance of antimicrobial resistance determinants. These functional profiles were consistent with the taxonomic differences previously identified in the same cohort and generate hypotheses regarding microbiome functions that may contribute to colonisation clearance. Because these findings are based on gene-content analysis, they reflect predicted functional potential rather than direct evidence of metabolic activity. Further multi-omics and experimental studies are required to validate these observations.

## 1. Introduction

Multidrug-resistant Gram-negative bacilli, including carbapenem-resistant Enterobacterales (CRE), are recognised by the World Health Organization (WHO) as a critical priority for research and antimicrobial development, representing a major global health threat [1]. In most cases, systemic CRE infection is preceded by asymptomatic intestinal colonisation, which constitutes the primary reservoir for subsequent infection and transmission [2,3].

Among CRE, OXA-48-producing Enterobacterales are of particular clinical relevance because OXA-48 is a class D carbapenemase that is widely disseminated in Europe, especially in Mediterranean countries, including Spain. OXA-48-producing isolates represent the predominant carbapenemase type in our institution and were therefore selected to minimise microbiological heterogeneity and enable a more consistent evaluation of microbiota-associated factors related to persistent carriage and spontaneous decolonization [4].

Unlike many other multidrug-resistant pathogens, carbapenem-resistant Enterobacterales establish persistent gastrointestinal colonisation, with the gut serving as their primary ecological reservoir and the main source of subsequent infection and transmission [4]. In contrast, pathogens such as *Pseudomonas aeruginosa*, *Acinetobacter baumannii*, and *Staphylococcus aureus* primarily colonise other body sites or environmental reservoirs. These differences make CRE particularly suitable for investigating gut microbiota-associated mechanisms of colonisation resistance and spontaneous decolonisation.

A healthy gut is characterised by both structural and functional integrity, including a balanced and diverse microbial composition [5]. Beyond its metabolic functions, the gut microbiota plays a critical role in maintaining epithelial barrier integrity, immune homeostasis, and protection against pathogen colonisation [6]. Colonisation resistance relies on two interdependent components: the gut microbiota and the host immune system [7]. The microbiota provides direct protection by occupying ecological niches, competing for nutrients, producing bacteriocins, mediating contact-dependent inhibition, and generating bioactive metabolites that modulate both microbial ecology and host physiology [8]. The host contributes through physical and chemical mucosal barriers, as well as innate and adaptive immune responses [9]. Disruptions in microbiota composition or function may compromise these protective mechanisms, thereby increasing susceptibility to infection [10,11,12].

Existing studies have primarily focused on taxonomic alterations associated with CRE colonisation and decolonisation, whereas less attention has been paid to the functional potential encoded within these microbial communities. Although taxonomic composition provides valuable ecological information, microorganisms with different taxonomic identities may contribute to similar biological functions, making functional profiling an important complementary approach for understanding microbiome-associated phenotypes. Consequently, characterising the functional potential of microbial communities may provide additional biological context for taxonomic signatures associated with CRE persistence or clearance.

Studies in other enteric pathogens have demonstrated that gut microbiota composition and function contribute to colonisation resistance. In *Clostridioides difficile* infection, disruption of the gut microbiota is associated with loss of colonisation resistance, whereas recovery of short-chain fatty acid (SCFA)-producing bacteria and restoration of microbial metabolic functions accompany microbiota recovery and pathogen clearance [13,14]. Similarly, studies of extended-spectrum β-lactamase (ESBL)-producing Enterobacterales have identified gut microbiome signatures associated with persistent carriage and spontaneous clearance [15]. Together, these findings suggest that microbial functional potential may be as important as taxonomic composition in determining colonisation outcomes. However, despite these advances, the functional characteristics associated with spontaneous decolonisation of carbapenem-resistant Enterobacterales remain poorly understood.

In a previous shotgun metagenomic study of this cohort, we identified distinct taxonomic profiles associated with spontaneous decolonisation and persistent CRE carriage [12]. However, whether these taxonomic differences are accompanied by differences in microbiome functional potential remains unknown.

This study aimed to characterise the predicted functional potential of the gut microbiota by analysing the abundance of KEGG orthologues in patients with persistent CRE carriage (Col) and spontaneous decolonisation (DeCol). Specifically, we sought to determine whether the taxonomic signatures previously identified in this cohort were accompanied by distinct functional profiles and metabolic pathway enrichments. The study was designed as a hypothesis-generating functional analysis based on metagenomic gene content rather than a direct assessment of microbial activity or metabolite production.

## 2. Results

A total of 37 patients were included, comprising 14 in the Col group and 23 in the DeCol group. All isolates from Col patients produced the OXA-48 carbapenemase, and 13 of 14 (93%) were identified as *Klebsiella pneumoniae*. The median duration of colonisation in the Col group was 14 months.

### 2.1. Differential Orthologue Abundance Analysis

A differential abundance analysis of KEGG orthologues was performed using DESeq2 comparing the DeCol and Col groups. A total of 231 orthologues met the predefined significance threshold (*p*_ad_ < 0.05 and |log_2_FC| > 1.5) (Supplementary Material S1). Each of these orthologues was first examined individually according to its KEGG annotation, known biological function, and involvement in microbial processes. Subsequently, orthologues with related biological functions or participating in common biological processes were grouped together to facilitate biological interpretation. From these groups, 37 representative orthologues were selected for detailed discussion because they provided biologically coherent functional signatures that were relevant to the patterns observed in our cohort and offered functional context for the taxonomic differences previously identified [12]. These included eight orthologues potentially associated with antimicrobial resistance and stress-response functions, eight related to substrate utilisation and metabolic pathways, eight linked to motility, biofilm formation and quorum sensing, and thirteen associated with genomic defence and toxin–antitoxin systems.

Applying more stringent filtering criteria (*p*_adj_ < 0.01 and |log_2_ FC| > 1.5), 99 orthologues remained in the list (Table 1).

**Figure 1:**
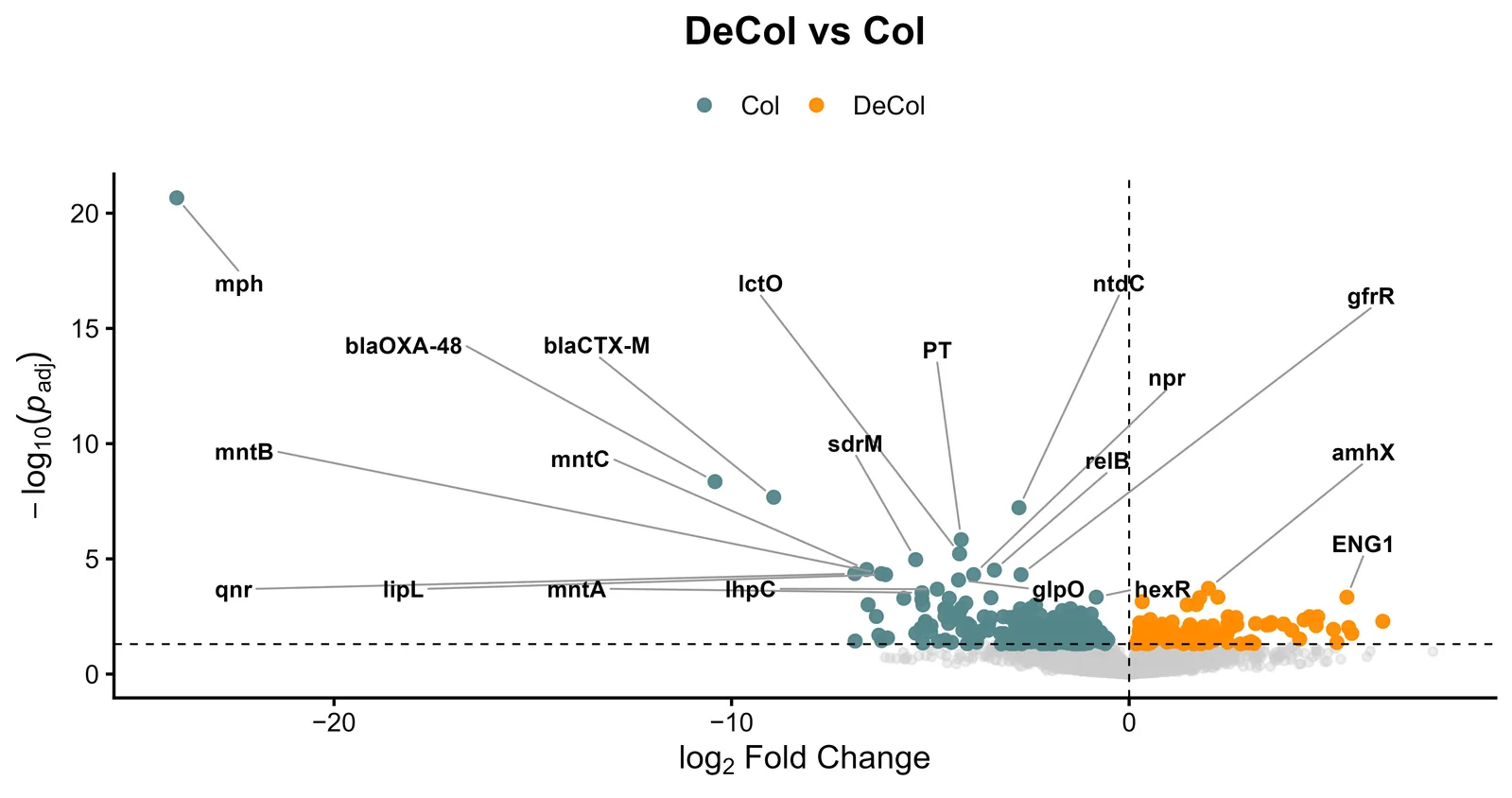
The 20 most abundant orthologues with the highest statistical significance in Col vs. DeCol groups.

In addition, Figure 1 shows the 20 most abundant orthologues with the highest statistical significance. To assess the robustness of our functional profiling, a sensitivity analysis was conducted using LinDA (Supplementary Material S2). Of the 231 significant orthologues identified in the primary analysis, 95 maintained their statistical significance under this framework. Furthermore, 89% of the biologically relevant orthologues were consistent between LinDA and DESeq2. These results support the robustness of the detected orthologue-level associations.

### 2.2. Orthologue Level by Functional Domain

The results presented below describe differences in predicted functional potential inferred from KEGG orthologue abundance and do not represent direct measurements of gene expression, metabolite production or pathway activity.

Subsequently, significant orthologues were interpreted according to their KEGG annotations and mapped onto representative metabolic pathways to facilitate biological interpretation. The pathways presented below were selected because they contained multiple differentially abundant orthologues and provided functional context for microbial taxa previously associated with persistent carriage or spontaneous decolonisation in this cohort [12]. Four major categories of functional signatures were identified.

#### 2.2.1. Antimicrobial Resistance and Stress-Adaptive Responses

We identified a higher abundance of several antimicrobial resistance-associated orthologues in Col patients, including class D beta-lactamases such as *bla*OXA-48 (KEGG orthologue K18976, log_2_ FC −10.42, *p*_adj_ = 4.45 × 10^−09^) and dihydrofolate reductase variants (DHFR; K18591, log_2_ FC = −6.22, *p*_adj_= 3.51 × 10^−02^).

In addition to these classical antimicrobial resistance determinants, Col patients showed a higher abundance of orthologues annotated as genes involved in stress-response and metabolic pathways, including manganese transporters (MntC/ABC transporters; K19975, log_2_ FC −6.60, *p*_adj_ = 2.89 × 10^−05^). Orthologues encoding amino acid transaminases and secretion-system components (Dot/lcm types IV and VI; K12218, log_2_ FC = −4.28, *p*_adj_ = 2.47 × 10^−03^) were also overrepresented in the Col group. Furthermore, several orthologues mapped to KEGG pathways related to redox-associated processes, including glutaredoxins (K06191, log_2_ FC = −1.82, *p*_adj_ = 1.41 × 10^−02^), glutathione reductase (K00383, log_2_ FC = −2.24, *p*_adj_ = 2.73 × 10^−03^) and peroxiredoxins (K04063, log_2_ FC = −2.61, *p*_adj_ = 2.21 × 10^−03^).

**Figure 2:**
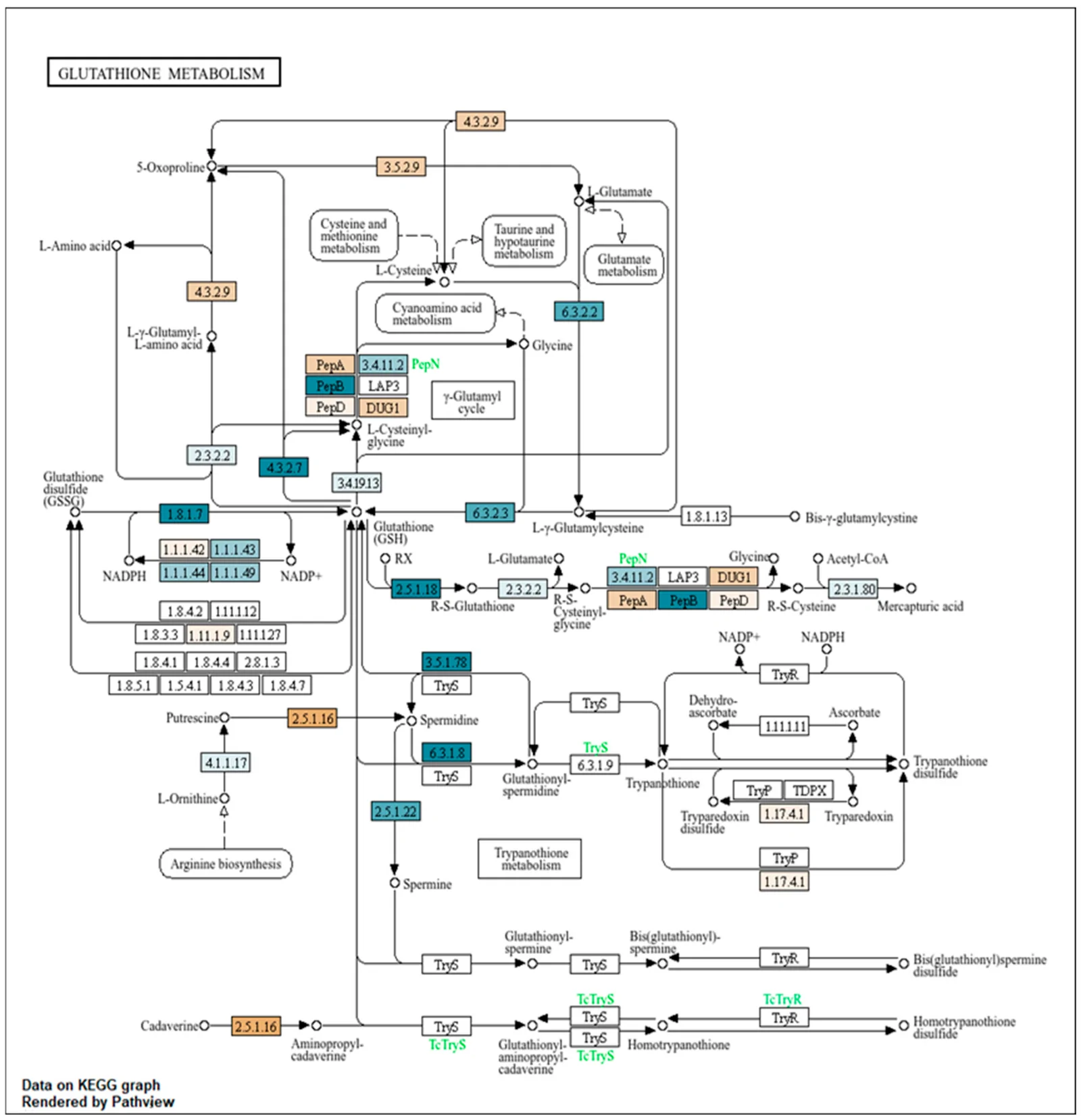
Glutathione metabolism pathway (map00480). The colour gradient indicates the log_2_ fold change (log_2_FC) of each specific gene. Pathway visualisations include all orthologues detected in the selected KEGG pathway and are provided to facilitate biological interpretation. Differential abundance was assessed separately using DESeq2, with significance defined as an adjusted *p*-value (padj) < 0.05 and |log2FC| > 1.5. Blue colours indicate underrepresentation in DeCol patients compared to Col (enzyme 1.8.1.7 was significantly underrepresented in the DeCol group), and orange colours indicate overrepresentation. To study the Kegg metabolic pathway in more detail, consult the website https://www.kegg.jp/pathway/map00480 (accessed on 14 July 2026).

To facilitate interpretation of these orthologue enrichment patterns, representative KEGG pathways containing multiple significant orthologues were examined. The cationic antimicrobial peptide resistance pathway (map01503), ABC transporters (map02010) (Supplementary Material S3) and glutathione metabolism (map00480) (Figure 2) were selected because they incorporated several of the differentially abundant genes identified in this category and provided functional context for taxa associated with persistent carriage in our previous study [12].

The ABC transporter pathway included multiple genes potentially associated with antimicrobial resistance, stress responses and nutrient acquisition. Within the glutathione metabolism pathway (Figure 2), several orthologues annotated as glutathione reductase (K00383, log_2_ FC −2.24, *p*_adj_ = 2.73 × 10^−03^) and other orthologues related to oxidative stress, such as lipoyl-dependent peroxiredoxin (K04063, log_2_ FC −2.61, *p*_adj_ = 2.21 × 10^−03^), NADH peroxidase (K05910, log_2_ FC −3.91, *p*_adj_ = 4.83 × 10^−05^), and glutaredoxin-like protein NrdH (K06191, log_2_ FC −1.82, *p*_adj_ = 1.41 × 10^−02^), were overrepresented in Col patients. These orthologues were differentially abundant between the two study groups within the glutathione metabolism pathway.

#### 2.2.2. Metabolic Pathway Signatures and Substrate Utilisation Potential

DeCol patients exhibited an increased abundance of orthologues mapping to pathways related to short-chain fatty acid (SCFA) metabolism, including acetate-, propionate- and butyrate-associated pathways. Higher abundance was also observed for genes involved in carbohydrate utilisation and substrate processing, including methylmalonyl-CoA carboxyltransferase 1.3S (K17490, log_2_ FC = 1.75, *p*_adj_ = 1.23 × 10^−2^), 6-carboxyhexanoate-CoA ligase (K01906, log_2_ FC 1.53, *p*_adj_ = 7.27 × 10^−3^) and glutamyl endopeptidase (K01318, log_2_ FC = 5.14, *p*_adj_ = 1.15 × 10^−2^).

**Figure 3:**
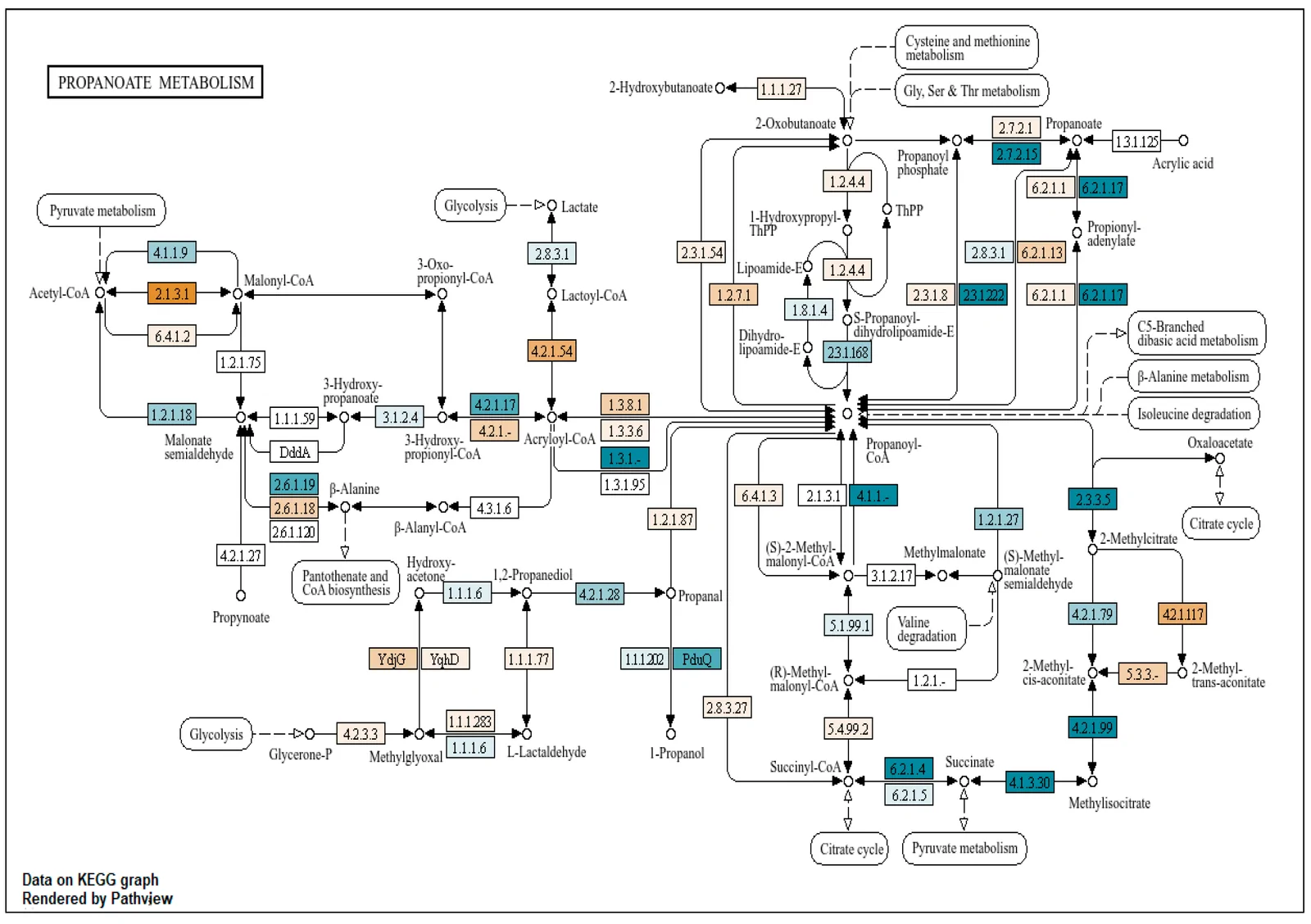
Propanoate metabolism pathway (map00640). The colour gradient indicates the log_2_ fold change (log_2_FC) of each specific gene. Pathway visualisations include all orthologues detected in the selected KEGG pathway and are provided to facilitate biological interpretation. Differential abundance was assessed separately using DESeq2, with significance defined as an adjusted *p*-value (padj) < 0.05 and |log2FC| > 1.5. Blue colours indicate underrepresentation in DeCol patients compared to Col, and orange colours indicate overrepresentation (enzyme 2.1.3.1 was significantly overrepresented in the DeCol group). To study the Kegg metabolic pathway in more detail, consult the website https://www.kegg.jp/pathway/map00640 (accessed on 14 July 2026).

The propanoate metabolism pathway (Figure 3) contained several orthologues that were overrepresented in DeCol patients: methylmalonyl-CoA carboxyltransferase 1.3S (K17490, log_2_ FC = 1.75, *p*_adj_ = 1.23 × 10^−2^), 6-carboxyhexanoate-CoA ligase (K01906, log_2_ FC 1.53, *p*_adj_ = 7.27 × 10^−3^) and glutamyl endopeptidase (K01318, log_2_ FC = 5.14, *p*_adj_ = 1.15 × 10^−2^). The propanoate metabolism pathway contained several differentially abundant orthologues and was selected as a representative pathway for visualisation.

**Figure 4:**
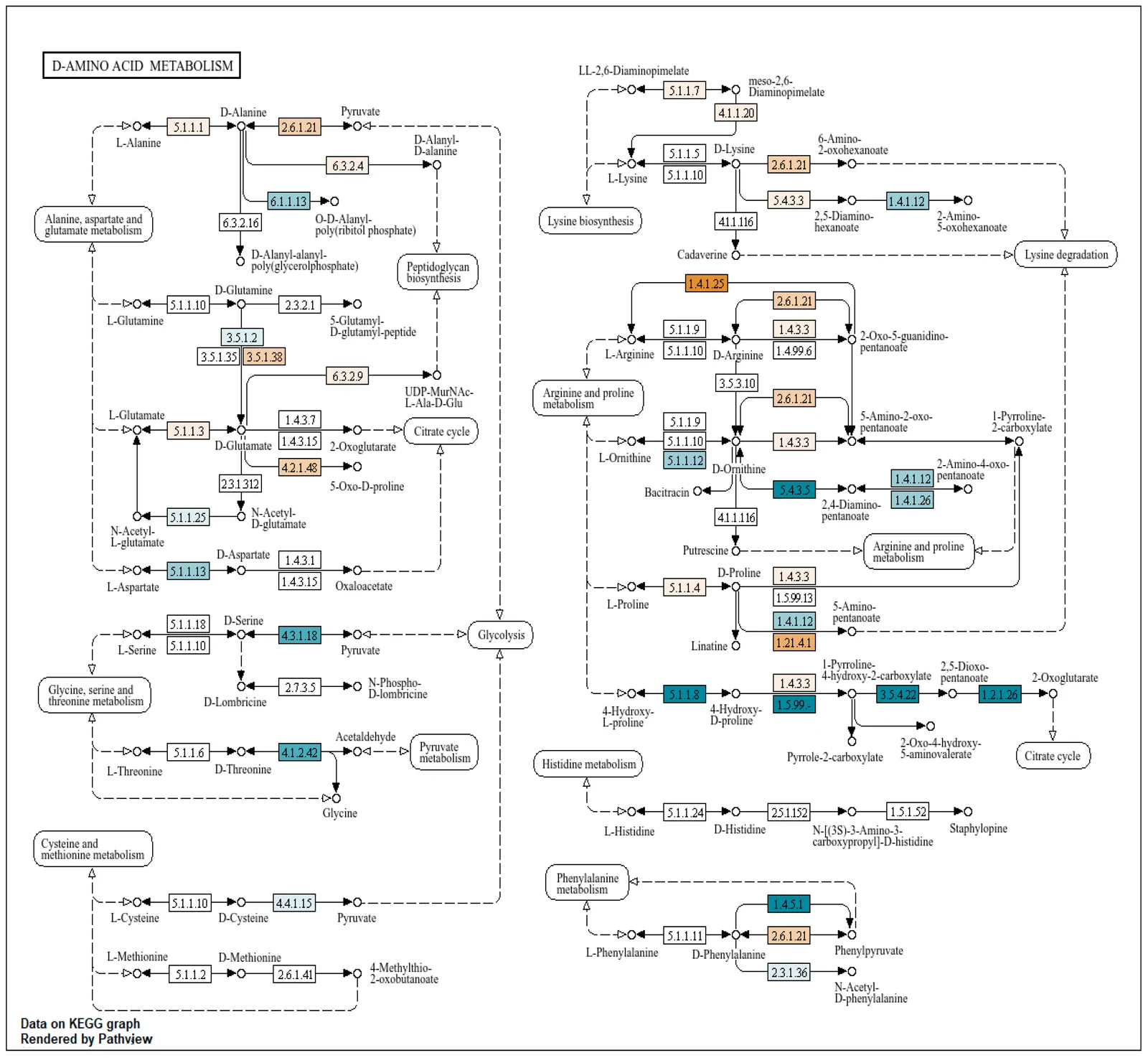
D-amino acid metabolism pathway (map00470). The colour gradient indicates the log_2_ fold change (log_2_FC) of each specific gene. Pathway visualisations include all orthologues detected in the selected KEGG pathway and are provided to facilitate biological interpretation. Differential abundance was assessed separately using DESeq2, with significance defined as an adjusted *p*-value (padj) < 0.05 and |log2FC| > 1.5. Blue colours indicate underrepresentation in DeCol patients compared to Col (enzymes 3.5.4.22, 1.5.99 and 1.2.1.26 were significantly underrepresented in the DeCol group), and orange colours indicate overrepresentation. To study the Kegg metabolic pathway in more detail, consult the website https://www.kegg.jp/pathway/map00470 (accessed on 14 July 2026).

**Figure 5:**
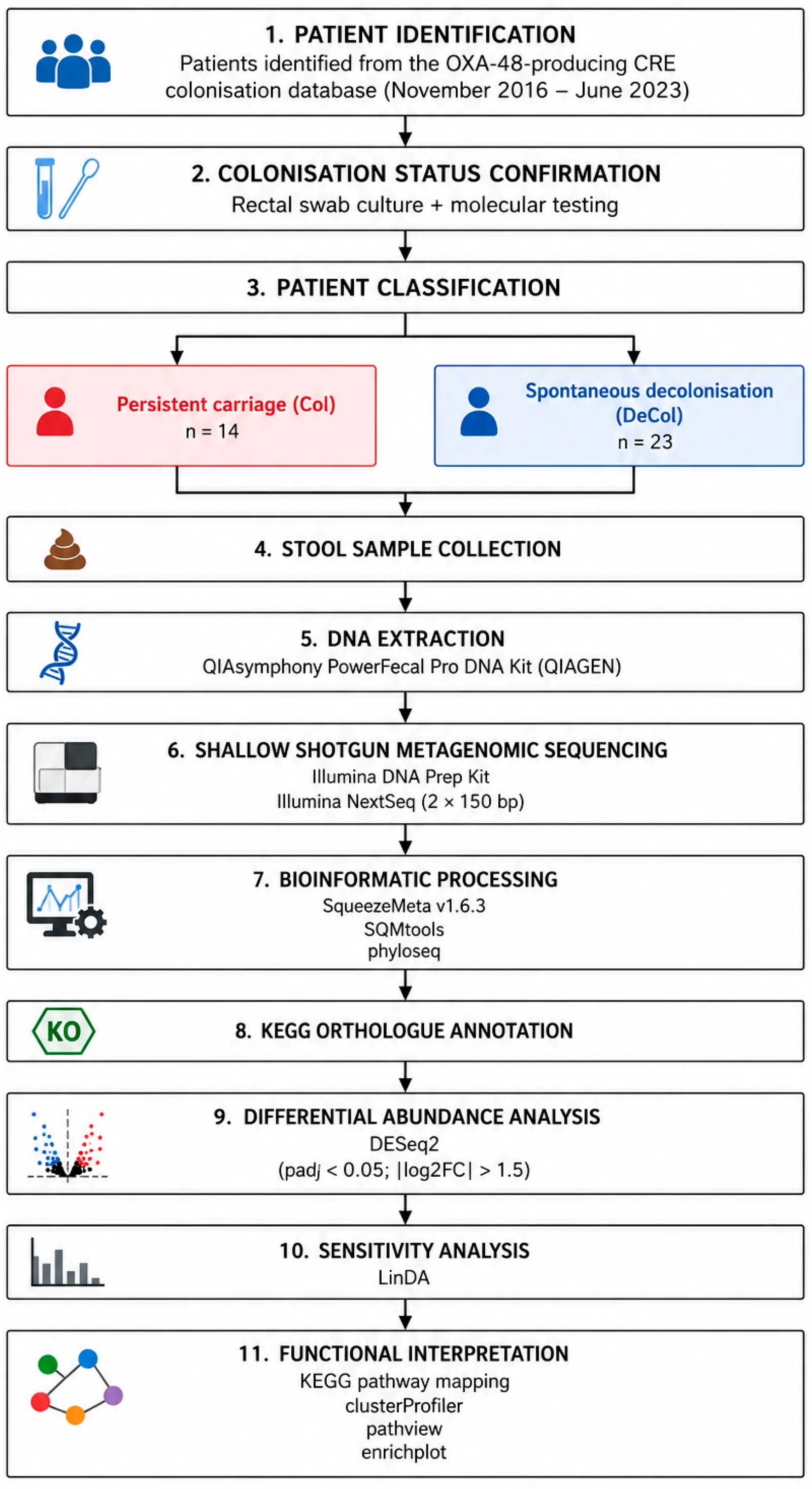
Workflow of the study design and analytical pipeline. Patients colonised with OXA-48-producing carbapenem-resistant Enterobacterales (CRE) were classified as persistent carriers (Col) or spontaneously decolonised (DeCol). Stool samples underwent genomic DNA extraction, shallow shotgun metagenomic sequencing, bioinformatic processing, KEGG orthologue annotation, differential abundance analysis using DESeq2, sensitivity analysis using LinDA, and biological interpretation through KEGG pathway mapping.

In contrast, pathways for D-amino acid metabolism (Figure 4) and biosynthesis of various antibiotics (Figure 5) contained several orthologues that were more abundant in Col patients. These orthologues were annotated as enzymes involved in amino acid transformation and alternative carbon and nitrogen utilisation pathways according to KEGG classification. These included the 1-pyrroline-4-hydroxy-2-carboxylate deaminase gene (K21062, log_2_ FC = −4.83, *p*_adj_ = 2.06 × 10^−4^; represented as 3.5.4.22) and the beta subunit of D-hydroxyproline dehydrogenase (K21061, log_2_ FC = −2.99, *p*_adj_ = 3.51 × 10^−3^, represented as 1.5.99). Additional genes of enzymes overrepresented in the Col group included the 2,5-dioxopentanoate dehydrogenase gene (K13877, log_2_ FC = −5.12, *p*_adj_ = 5.17 × 10^−3^, represented as 1.2.1.26) and a putative aminopeptidase gene (K20609, log_2_ FC = −2.53, *p*_adj_ = 3.54 × 10^−3^).

DeCol patients also exhibited increased abundance of genes involved in broader nutrient acquisition and substrate processing. These included orthologues associated with proteolytic activity, such as glutamyl endopeptidase (K01318, log_2_ FC = 5.14, *p*_adj_ = 1.15 × 10^−2^), and enzymes involved in carbohydrate and polysaccharide metabolism, including phosphomannomutase (K17497, log_2_ FC = 6.38, *p*_adj_ = 5.13 × 10^−3^). Together, these findings demonstrate differences in the abundance of orthologues annotated to substrate utilisation pathways between groups.

#### 2.2.3. Motility, Biofilm Formation, and Quorum Sensing

DeCol patients showed a higher abundance of orthologues annotated as components of motility-, adhesion- and polysaccharide-related pathways, including genes associated with pilus-mediated motility (K02660, log_2_ FC = 4.54, *p*_adj_ = 3.23 × 10^−3^ and K12069, log_2_ FC = 4.71, *p*_adj_ = 7.92 × 10^−3^), flagellar biosynthesis activator protein and rhamnosyltransferases (K12991, log_2_ FC = 3.46, *p*_adj_ = 7.44 × 10^−3^).

The DeCol group also exhibited overrepresentation of orthologues related to polysaccharide synthesis, and additional orthologues annotated as PelG (K21012, log_2_ FC = 1.77, *p*_adj_ = 4.87 × 10^−4^), quorum-sensing regulator LiR/HapR and diguanylate cyclase (K21020, log_2_ FC = 1.69, *p*_adj_ = 9.57 × 10^−4^) were also overrepresented in DeCol patients.

Conversely, orthologues annotated to *quorum sensing (map02024)* and community-level biofilm formation pathways were more abundant in the Col group, including the AI-2 transport system permease (K10557, log_2_ FC = −2.03, *p*_adj_ = 6.51 × 10^−3^, represented as LsrD) and ATP-binding protein (K10558, log_2_ FC = −1.86, *p*_adj_ = 5.55 × 10^−3^, represented as LsrA) and the substrate-binding protein (orthologue K10555; log_2_ FC −1.86, *p*_adj_ = 1.53 × 10^−2^; represented as LsrC).

#### 2.2.4. Genomic Defence and Toxin–Antitoxin Systems

Several orthologues associated with genomic defence, mobile genetic element control and toxin–antitoxin systems differed significantly between groups. Most of these functions were overrepresented in Col patients, including CRISPR-associated proteins Cas5e (K18841, log_2_ FC = −3.54, *p*_adj_ = 1.25 × 10^−2^) and Cas6e (K18842, log_2_ FC = −2.89, *p*_adj_ = 3.64 × 10^−2^) and the CRISPR-associated endoribonuclease Cas2 (K03487, log_2_ FC = −1.66, *p*_adj_ = 8.18 × 10^−3^). Additional orthologues linked to genome maintenance and defence against foreign genetic elements were also enriched in the Col group, including K16135 (log_2_ FC = −2.14, *p*_adj_ = 1.33 × 10^−2^), K18918 (log_2_ FC = −3.39, *p*_adj_ = 3.07 × 10^−5^), K19505 (log_2_ FC = −2.72, *p*_adj_ = 4.83 × 10^−5^), K10917 (log_2_ FC = −2.85, *p*_adj_ = 1.18 × 10^−2^), K21828 (log_2_ FC = −3.54, *p*_adj_ = 7.27 × 10^−3^), K18320 (log_2_ FC = −4.63, *p*_adj_ = 3.11 × 10^−3^) and the toxin–antitoxin system component K07498 (log_2_ FC = −4.22, *p*_adj_ = 1.47 × 10^−6^).

Conversely, a smaller subset of orthologues was overrepresented in DeCol patients, including K21492 (log_2_ FC = 5.60, *p*_adj_ = 1.72 × 10^−2^), K12069 (log_2_ FC = 4.71, *p*_adj_ = 7.92 × 10^−3^) and K11686 (log_2_ FC = 4.40, *p*_adj_ = 4.46 × 10^−3^). Overall, orthologues related to CRISPR-Cas systems, toxin–antitoxin modules and genome-associated functions were differentially distributed between persistent carriage and spontaneous decolonisation.

## 3. Discussion

The findings of this work complement the results of our previous study [12]. In our cohort, spontaneous decolonisation of carbapenem-resistant Enterobacterales (CRE) was associated with distinct taxonomic signatures, like *Suterella*, *Roseburia faecis* and *Eubacterium ventriosum*. Meanwhile, CRE colonisation was associated with a higher abundance of *Ruthenibacterium lactatiformans*, *Klebsiella pneumoniae* and *Lactobacillus crispatus*. In the present work, we extend these observations by showing that these taxonomic differences are accompanied by distinct KEGG-based functional signatures and differences in predicted functional potential. Taken together, both studies suggest that spontaneous decolonisation is associated not only with compositional differences but also with functional profiles that are compatible with biological processes previously linked to colonisation resistance [8,16].

Persistent colonisation and spontaneous decolonisation were associated with two contrasting predicted functional profiles inferred from KEGG orthologue abundance in our cohort. The orthologue enrichment patterns observed in Col patients were compatible with pathways previously associated with stress responses and dysbiosis, whereas DeCol patients exhibited enrichment of orthologues mapping to pathways related to carbohydrate metabolism and short-chain fatty acid (SCFA)-associated functions. Similar functional signatures have been reported in studies of CRE and ESBL-producing Enterobacterales carriers, where differences in antimicrobial resistance determinants and stress-associated pathways have been observed [15,17]. This stress-adapted phenotype has also been described in other dysbiosis-associated conditions, particularly in *Clostridioides difficile* infection (CDI). In CDI, disruption of the microbiota leads to loss of colonisation resistance, expansion of facultative anaerobes, and increased oxidative and inflammatory metabolism, which favours pathogen persistence [13,14].

The higher abundance of antimicrobial resistance-associated orthologues, including *bla*OXA-48 and DHFR variants, in Col patients is likely explained by the persistent intestinal colonisation with OXA-48-producing Enterobacterales, which inherently harbour these resistance determinants. Previous healthcare-associated selective pressures, including antibiotic exposure, may also contribute to the maintenance of these genes. However, because our analysis is based on metagenomic gene content, these findings reflect differences in gene abundance rather than active gene expression or resistance mechanisms.

In our Col patients, the enrichment of orthologues annotated to redox defence pathways may indicate a greater representation of genes associated with oxidative stress-related functions, rather than direct evidence of increased oxidative stress responses. Similar orthologues have previously been described in bacterial populations adapted to inflammatory or antibiotic-exposed environments [18,19]. At the same time, the enrichment of arachidonic acid metabolism, steroid degradation and aminobenzoate degradation further suggests differences in the predicted utilisation of host-associated metabolites between groups [8,20]. The overrepresentation of orthologues annotated to biofilm formation and quorum sensing pathways is consistent with predicted functional profiles previously reported in persistent Enterobacterales colonisation. However, these findings reflect gene abundance rather than direct evidence of biofilm formation or quorum-sensing activity. Biofilm-associated communities are known to enhance tolerance to antibiotics and host defences, and have been linked to chronic colonisation in both CRE and ESBL-producing Enterobacterales [17,21]. Col patients also showed enrichment of amino acid catabolism pathways, particularly those linked to host-derived substrates such as proline and hydroxyproline. Similar orthologue enrichment patterns have been reported in dysbiotic and inflamed gut environments, including those observed during CDI [14,22].

These functional signatures provide biological context for the taxonomic findings reported in our previous study [12]. In particular, the expansion of *Klebsiella pneumoniae* has been consistently associated with an increase in defence systems against oxidative stress, including pathways such as glutathione metabolism and redox enzymes, which have previously been associated with adaptation to oxidative and inflammatory conditions [23]. Furthermore, *K. pneumoniae* exhibits a remarkable capacity for biofilm formation and regulation via quorum sensing, contributing to its intestinal persistence and antimicrobial resistance [24]. These observations are consistent with the orthologue enrichment patterns detected in the Col group. On the other hand, species such as *Parabacteroides* spp. have been linked to the transformation of lipid and aromatic metabolites, which could connect with the enrichment of pathways such as arachidonic acid metabolism, steroid degradation, and aminobenzoate degradation [25]. In this context, *Lactobacillus* spp. have demonstrated the ability to resist oxidative stress and modulate intestinal redox balance, contributing to survival in altered environments [26].

In contrast, DeCol patients exhibited enrichment of orthologues mapping to SCFA-associated pathways together with pathways related to carbohydrate utilisation and substrate processing. Although SCFAs have been implicated in these biological processes, our data do not demonstrate SCFA production or metabolic activity, but rather differences in the abundance of genes annotated to SCFA-related pathways [16,27]. Although SCFA concentrations were not measured in the present study, the enrichment of genes mapping to SCFA-related pathways is consistent with these previously described functions. From a clinical standpoint, these functional signatures are compatible with microbiome configurations that have previously been associated with colonisation resistance and have been consistently linked to protection against pathogens such as *C. difficile* [13]. Importantly, similar functional patterns have been reported in studies of microbiota recovery after CDI or antibiotic exposure, where restoration of SCFA-producing bacteria and carbohydrate metabolism pathways correlates with pathogen clearance [13,14]. In the context of ESBL and CRE colonisation, recent studies also highlight that individuals who clear colonisation tend to recover a microbiota enriched in fibre-fermenting, SCFA-producing taxa, supporting the hypothesis that recovery of specific microbiome-associated metabolic functions may contribute to decolonisation [13,17].

Another relevant finding in the DeCol group was the enrichment of genes related to microbial interaction, motility and regulated adhesion. These features may indicate differences in microbial interaction and colonisation-related functions represented within the microbiota and resource utilisation [8]. These observations are consistent with ecological models of colonisation resistance in which multiple microbial functions contribute to community stability [7,8].

Another noteworthy finding was the differential distribution of orthologues involved in genomic defence and toxin–antitoxin systems. Contrary to what might be expected in a stable microbiota, several CRISPR-associated proteins (Cas2, Cas5e and Cas6e) together with multiple genes involved in genome protection and toxin–antitoxin modules were enriched in the Col group. CRISPR-Cas systems constitute adaptive immune mechanisms that protect bacteria against bacteriophages and foreign DNA, while toxin–antitoxin systems contribute to genome stabilisation, stress tolerance and persistence under adverse environmental conditions. Recent studies have shown that these systems are frequently enriched in bacterial populations exposed to intense ecological competition, phage predation and horizontal gene transfer, conditions commonly observed in dysbiotic microbiomes dominated by opportunistic pathogens [28,29,30]. In addition, toxin–antitoxin modules have been linked to plasmid maintenance and long-term persistence of antimicrobial resistance determinants, including carbapenemase-encoding plasmids [31]. Therefore, the enrichment of these orthologues may reflect differences in the ecological and genetic contexts represented within the microbiota of persistently colonised patients. However, because these data are based on gene abundance alone, the biological significance of these findings remains uncertain and warrants further investigation.

The taxonomic signatures previously identified in DeCol patients may provide a biological explanation for some of the functional profiles observed in the present study. In particular, *Roseburia faecis* and *Eubacterium ventriosum* are recognised butyrate-producing species and may contribute to the enrichment of orthologues mapping to SCFA-associated pathways. All of this contributes to functional signatures that have previously been associated with SCFA production and intestinal homeostasis [32]. Butyrate, produced via fermentation of dietary fibre in the colon, serves as a primary energy source for colonocytes, supports gut barrier integrity, promotes microbiota homeostasis, and possesses anti-inflammatory properties [33]. Additionally, the presence of *Sutterella* spp. has been associated with host–microbe interactions at the mucosal interface, potentially providing biological context for the enrichment of orthologues annotated as motility- and adhesion-related functions, which are essential for microbial colonisation and persistence [34]. Taken together, these findings suggest that the taxonomic and functional signatures identified in this cohort are interconnected and may represent complementary aspects of the same ecological configuration. Rather than acting in isolation, the taxa identified in DeCol and Col patients may contribute to distinct functional potentials represented within the microbiota. Further studies integrating metagenomics with transcriptomics, metabolomics and experimental validation will be required to determine the biological relevance of these associations.

An additional consideration when interpreting these findings is that KEGG orthologues are functional annotation units rather than pathway-specific markers. Individual orthologues may participate in multiple biological processes or metabolic pathways depending on the organism and its physiological context. Consequently, the pathway enrichments reported here should be interpreted as providing functional context for the observed gene-content differences rather than indicating activation of specific biological processes.

From a clinical perspective, the functional signatures identified in this study may have potential diagnostic and therapeutic implications. If validated in larger prospective cohorts, orthologues associated with antimicrobial resistance, oxidative stress adaptation, or SCFA-related metabolism could serve as biomarkers to distinguish persistent CRE carriage from spontaneous decolonisation. Likewise, the enrichment of pathways linked to carbohydrate metabolism and SCFA production in decolonised patients supports the rationale for microbiota-targeted interventions, including dietary modulation, probiotics, or faecal microbiota transplantation, aimed at restoring colonisation resistance. However, these potential applications remain speculative, as our findings are based on predicted functional potential rather than direct measurements of microbial activity, and therefore require validation using complementary multi-omics approaches and experimental studies.

This study has several limitations. First, it is a single-centre study with a relatively small sample size (n = 37), which may limit the generalisability of the findings. It also includes only patients colonised by OXA-48-producing Enterobacterales. Second, the case–control design precludes causal inference, allowing only the identification of associations between microbiota functional profiles and colonisation status. Third, functional inferences are based on KEGG annotations and therefore reflect metabolic potential rather than actual gene expression or activity. Consequently, the pathways discussed throughout this study should be interpreted as indicators of predicted functional potential and hypothesis-generating associations rather than direct evidence of active biological processes. Finally, it is also difficult to establish the weight of the history of previous admissions or antibiotic treatments of the Col group described in the previous paper [12].

## 4. Materials and Methods

We conducted a single-centre case–control study involving adult patients colonised with OXA-48-producing CRE. Patients were identified from a CRE colonisation database covering the period between November 2016 and June 2023. During the week preceding microbiota sampling, all participants underwent rectal swabbing for molecular testing to confirm their colonisation status. No patient was under antibiotic treatment at the time the stool sample was collected. Based on these results, patients were classified as currently colonised (Col) or spontaneously decolonised (DeCol). All participants completed a standardised questionnaire assessing lifestyle and dietary habits, the data from which were incorporated into the analytical framework [12].

DeCol patients were defined as those with either three consecutive negative rectal swab cultures or two negative cultures combined with a negative molecular test, following a documented previous colonisation [35]. Patients were classified as Col if they had at least two positive samples and did not fulfil the criteria for decolonisation. All decolonisation events were considered spontaneous, as no targeted decolonisation protocol has been implemented at our institution.

### Sample Processing and Bioinformatic and Statistical Analysis

Genomic DNA was extracted from stool samples using the QIAsymphony PowerFecal Pro DNA Kit (QIAGEN, Hilden, Germany). The entire methodological description is set out in the previous paper [12]. Gut microbiota profiling was performed using shallow shotgun metagenomic sequencing (yielding approximately 5.5 million reads per sample). Libraries were prepared with the Illumina DNA Prep Kit (Illumina Inc., San Diego, CA, USA) according to the manufacturer’s protocol and sequenced on an Illumina NextSeq platform (P2 cartridge, 2 × 150 bp). The datasets generated and analysed during the current study are available in the European Nucleotide Archive (ENA) repository (accession numbers PRJEB111255, ERR16994728-ERR16994772).

Sequencing data were processed using the fully automated SqueezeMeta v1.6.3 pipeline [36] executed at the Galicia Supercomputing Centre (CESGA, Spain). Output files were imported into R (version 4.5.0) using the SQMtools package (v1.7.2) [37]. A phyloseq object was generated for downstream analyses with the phyloseq v1.54.2 R package [38]. Tables were formatted using the gtsummary v2.5.0 [39] and kableExtra v1.4.0 [40] R packages.

Functional profiling was performed by analysing the differential abundance of genes associated with the Kyoto Encyclopedia of Genes and Genomes (KEGG) database [41] to characterise differences in the predicted functional potential of the gut microbiota between DeCol and Col patients. Functional interpretations were based on gene-content profiles derived from metagenomic sequencing and therefore reflect inferred metabolic potential rather than direct measurements of pathway activity.

Differential abundance of individual genes was assessed using DESeq2 v1.50.2 [42] from the raw data. DESeq2 was selected as the primary differential abundance method because it directly models raw count data using a negative binomial distribution, provides robust normalisation and empirical Bayes estimation of dispersion parameters, and performs reliably in studies with relatively small sample sizes. These characteristics make it particularly suitable for shotgun metagenomic gene-count data such as the KEGG orthologue counts analysed in this study. Because microbiome datasets are inherently compositional, we additionally performed a sensitivity analysis using LinDA, a method specifically developed for microbiome differential abundance analysis. The high concordance between both approaches supports the robustness of our findings. A false discovery rate (FDR) adjusted *p*-value *p*_adj_ < 0.05 combined with an absolute log_2_ fold change (|log_2_ FC|) > 1.5 was considered statistically significant. Additionally, a more restrictive threshold of *p*_adj_ < 0.01 and |log_2_ FC| > 1.5 was applied to identify top-priority orthologues. To obtain graphical representations of the results, the significantly differential orthologues were subsequently subjected to an overrepresentation analysis with a significance threshold of *p*_adj_ < 0.05, using the following R packages: clusterProfiler v4.18.4 [43], DOSE v4.4.0 [44], pathview v1.50.0 [45], and enrichplot v1.30. [43]. Orthologues were first analysed individually and subsequently grouped into functional categories according to KEGG annotations. Representative pathways were selected when they contained multiple differentially abundant orthologues and provided biological context for the taxonomic signatures previously identified in the same cohort [12]. Pathway visualisations were used as an interpretative tool to summarise orthologue-level differences rather than as evidence of pathway activation. To ensure the robustness of our findings, a sensitivity analysis was subsequently conducted using LinDA v0.2.0 to validate the differentially abundant genes. For this complementary analysis, the model was applied to the raw abundance data after filtering out low-prevalence genes (present in <5% of the samples).

The software tools and databases used in this study are publicly available, and their official websites have been included in the corresponding references.

## 5. Conclusions

In conclusion, spontaneous CRE decolonisation was associated with distinct KEGG-based functional signatures and a lower abundance of antimicrobial resistance determinants. These functional profiles were consistent with the taxonomic differences previously identified in the same cohort and suggest differences in the predicted metabolic potential of the gut microbiota between persistent carriage and spontaneous decolonisation. In particular, DeCol patients exhibited enrichment of orthologues mapping to short-chain fatty acid (SCFA)-associated and carbohydrate-utilisation pathways, whereas Col patients showed a higher abundance of orthologues related to antimicrobial resistance, redox-associated processes and biofilm-related functions.

Because these findings are based on metagenomic gene-content analysis, they should be interpreted as indicators of predicted functional potential rather than direct evidence of biological activity. Nevertheless, they provide functional context for previously identified taxonomic signatures and generate testable hypotheses regarding microbiome-associated processes that may contribute to CRE persistence or clearance. Future studies integrating shotgun metagenomics with complementary multi-omics approaches, including metatranscriptomics and metabolomics, together with experimental validation using functional assays and in vitro or in vivo models, will be important to confirm the biological relevance of the identified genes and pathways and to determine whether these predicted functional signatures translate into active microbial metabolism and contribute to spontaneous CRE decolonisation.

## Figures and Tables

**Table 1:** Orthologues over- and under-represented in DeCol patients with respect to Col patients: log_2_ fold change (log_2_FC) > 1.5, adjusted *p*-value (*p*_adj_) < 0.01.

Pathway	KO_name	log_2_FC	*p*_adj_
K18976	beta-lactamase class D OXA-48	−27.1614	8.09 × 10^−50^
K18591	dihydrofolate reductase (trimethoprim resistance protein)	−25.1265	5.43 × 10^−24^
K21949	N-(2-amino-2-carboxyethyl)-L-glutamate synthase	22.2688	3.89 × 10^−18^
K06979	macrolide phosphotransferase	−26.2551	3.20 × 10^−17^
K18590	dihydrofolate reductase (trimethoprim resistance protein)	−26.1437	5.02 × 10^−15^
K20381	regulatory protein	−24.7134	2.50 × 10^−13^
K19060	TetR/AcrR family transcriptional regulator, macrolide resistance operon repressor	−24.2071	8.67 × 10^−13^
K18845	16S rRNA (guanine(1405)-N(7))-methyltransferase	−23.8977	1.76 × 10^−12^
K19061	macrolide resistance protein	−23.6424	3.11 × 10^−12^
K00967	ethanolamine-phosphate cytidylyltransferase	22.3099	1.23 × 10^−10^
K18653	3-dehydro-glucose-6-phosphate—glutamate transaminase	22.2438	1.32 × 10^−10^
K18543	choriolysin H	22.1715	1.45 × 10^−10^
K18767	beta-lactamase class A CTX-M	−10.3016	1.65 × 10^−10^
K22351	beta-lactamase class D OXA-209	22.0590	1.65 × 10^−10^
K18652	glucose-6-phosphate 3-dehydrogenase	−2.7130	9.80 × 10^−9^
K07498	putative transposase	−4.7116	2.88 × 10^−7^
K01318	glutamyl endopeptidase	7.7867	3.18 × 10^−5^
K14665	amidohydrolase	2.4104	3.70 × 10^−5^
K11686	chromosome-anchoring protein RacA	6.1495	9.21 × 10^−5^
K18935	MFS transporter, DHA2 family, multidrug resistance protein	−5.32248	0.00019
K19975	manganese transport system substrate-binding protein	−6.74772	0.00048
K21490	antitoxin YokJ	6.81343	0.00052
K21062	1-pyrroline-4-hydroxy-2-carboxylate deaminase [EC:3.5.4.22]	−5.18257	0.00056
K21492	antitoxin YqcF	8.08715	0.0007
K16509	regulatory protein spx	−2.41125	0.00087
K19505	sigma-54 dependent transcriptional regulator, gfr operon transcriptional activator	−2.23737	0.00115
K18589	dihydrofolate reductase (trimethoprim resistance protein) [EC:1.5.1.3]	−8.47016	0.00129
K12209	intracellular multiplication protein IcmE	−4.41206	0.00173
K17497	phosphomannomutase [EC:5.4.2.8]	7.46717	0.00202
K01180	endo-1,3(4)-beta-glucanase [EC:3.2.1.6]	5.0599	0.00205
K10558	AI-2 transport system ATP-binding protein	−1.76524	0.00249
K21012	polysaccharide biosynthesis protein PelG	1.71047	0.00337
K18130	TetR/AcrR family transcriptional regulator, transcriptional repressor NalC	6.50571	0.00337
K02760	cellobiose PTS system EIIB component [EC:2.7.1.196 2.7.1.205]	−1.5819	0.00343
K19220	peptidoglycan DL-endopeptidase CwlS [EC:3.4.-.-]	−2.86088	0.00549
K02240	competence protein ComFA	−1.73531	0.007
K05816	sn-glycerol 3-phosphate transport system ATP-binding protein [EC:7.6.2.10]	−1.60216	0.0073
K09985	uncharacterised protein	6.55133	0.00756
K04063	lipoyl-dependent peroxiredoxin [EC:1.11.1.28]	−2.56327	0.00825
K08151	MFS transporter, DHA1 family, tetracycline resistance protein	−2.20258	0.00997

## Data Availability

The data presented in this study are openly available in EMBL-EBI at https://www.ebi.ac.uk/ena/browser/view/PRJEB111255 (accessed on 14 July 2026), reference number PRJEB111255.

## Supplementary Materials

The following supporting information can be downloaded at: https://www.mdpi.com/article/10.3390/ijms27167092/s1.
