# Supplementary material for: KEGG-Based Functional Signatures Complement Taxonomic Profiles Associated with Spontaneous Decolonisation of Carbapenem-Resistant Enterobacterales
Source: Int J Mol Sci. 2026 Aug 7;27(16):7092. doi: 10.3390/ijms27167092 (PMC13512929; doi:10.3390/ijms27167092)
Supplement: Supplementary file 1 [file ijms-27-07092-s001.zip › Supplementary Material S3. ABC Transporters metabolic pathway (map02010).pdf]

ABC Transporters metabolic pathway (map02010). The color gradient indicates the log2 Fold Change of each specific gene. Blue colors indicate underrepresentation in DeCol patients compared to Col, and orange colors indicate overrepresentation.

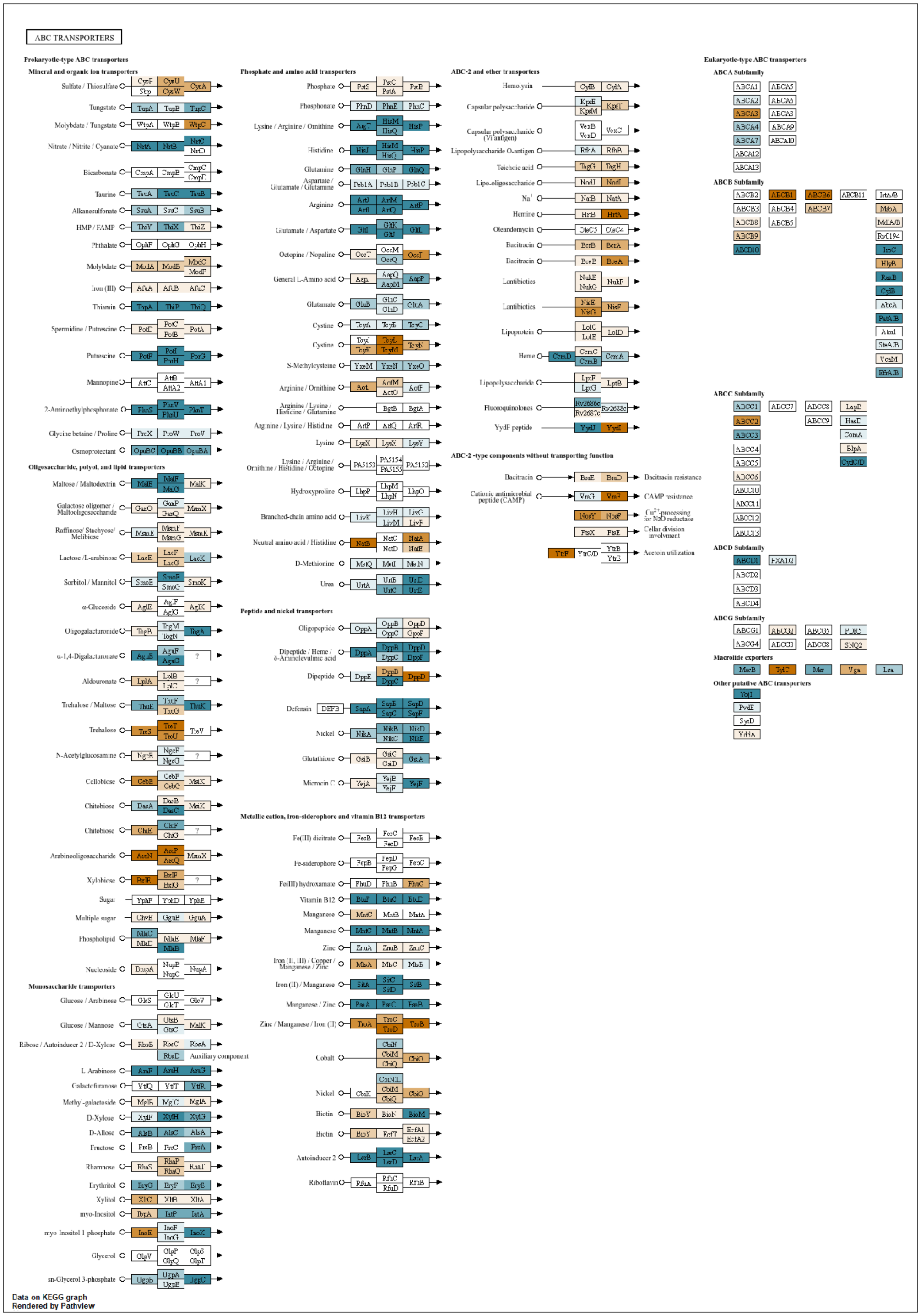
ABC TRANSPORTERS
Prokaryotic-type ABC transporters
Mineral and organic ion transporters
Phosphate and amino acid transporters
ABC-2 and other transporters
Eukaryotic-type ABC transporters
ABCA Subfamily
ABCB Subfamily
ABCC Subfamily
ABCD Subfamily
ABCG Subfamily
Macrolide exporters
Other putative ABC transporters
Oligosaccharide, polyol, and lipid transporters
Monosaccharide transporters
Peptide and nickel transporters
Metallic cation, iron-siderophore and vitamin B12 transporters
ABC-2-type components without transporting function
Data on KEGG graph
Rendered by Pathview
